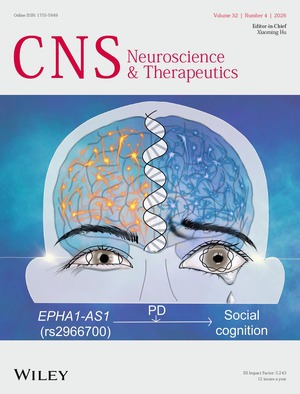# Front Cover

**DOI:** 10.1002/cns.70869

**Published:** 2026-04-08

## Abstract

The cover image is based on the article *Deciphering the Impact of EPHA1‐AS1 Gene Polymorphism on Social Cognition Deficits in Parkinson's Disease* by Yu‐Chen Lin et al., https://doi.org/10.1002/cns.70801.